# MAGI2 Gene Region and Celiac Disease

**DOI:** 10.3389/fnut.2019.00187

**Published:** 2019-12-19

**Authors:** Amaia Jauregi-Miguel, Izortze Santin, Koldo Garcia-Etxebarria, Ane Olazagoitia-Garmendia, Irati Romero-Garmendia, Maialen Sebastian-delaCruz, Iñaki Irastorza, Spanish Consortium for the Genetics of Celiac Disease, Ainara Castellanos-Rubio, Jose Ramón Bilbao

**Affiliations:** ^1^Department of Genetics, Physical Anthropology and Animal Physiology, Biocruces-Bizkaia Health Research Institute, University of the Basque Country (Universidad del País Vasco/Euskal Herriko Unibertsitatea), Leioa, Spain; ^2^Department of Biochemistry and Molecular Biology, Biocruces-Bizkaia Health Research Institute, University of the Basque Country (Universidad del País Vasco/Euskal Herriko Unibertsitatea), Leioa, Spain; ^3^CIBER in Diabetes and Associated Metabolic Diseases, Madrid, Spain; ^4^Department of Pediatrics, Biocruces-Bizkaia Health Research Institute, Cruces University Hospital, University of the Basque Country (Universidad del País Vasco/Euskal Herriko Unibertsitatea), Barakaldo, Spain; ^5^Ikerbasque, Basque Foundation for Science, Bilbao, Spain

**Keywords:** celiac disease, tight junction, association analysis, expression analysis, MAGI2, long non-coding RNA

## Abstract

Celiac disease (CD) patients present a loss of intestinal barrier function due to structural alterations in the tight junction (TJ) network, the most apical unions between epithelial cells. The association of TJ-related gene variants points to an implication of this network in disease susceptibility. This work aims to characterize the functional implication of TJ-related, disease-associated loci in CD pathogenesis. We performed an association study of 8 TJ-related gene variants in a cohort of 270 CD and 91 non-CD controls. The expression level of transcripts located in the associated SNP region was analyzed by RT-PCR in several human tissues and in duodenal biopsies of celiac patients and non-CD controls. (si)RNA-driven silencing combined with gliadin in the Caco2 intestinal cell line was used to analyze the implication of transcripts from the associated region in the regulation of TJ genes. We replicated the association of rs6962966^*^A variant [*p* = 0.0029; OR = 1.88 (95%1.24–2.87)], located in an intron of TJ-related MAGI2 coding gene and upstream of RP4-587D13.2 transcript, bioinformatically classified as a long non-coding RNA (lncRNA). The expression of both genes is correlated and constitutively downregulated in CD intestine. Silencing of lncRNA decreases the levels of MAGI2 protein. At the same time, silencing of *MAGI2* affects the expression of several TJ-related genes. The associated region is functionally altered in disease, probably affecting CD-related TJ genes.

## Introduction

Celiac disease (CD) is a chronic enteropathy, characterized by villous atrophy and inflammation of the intestinal mucosa, that develops in genetically predisposed individuals. The disease is caused by an inappropriate immune response to ingested gluten, a protein consisting of soluble gliadin and insoluble glutenin components that is found mostly in wheat, but also in barley, rye and oat. There is evidence supporting that various environmental factors, such as repeated gastrointestinal infections (1, 2) or early infant feeding (3, 4) may also contribute to triggering the disease. A lifelong gluten-free diet (GFD) is the only effective treatment for CD that achieves complete remission of the symptoms.

CD is a complex genetic disorder, and multiple genes contribute to disease risk. The most important genetic determinants, Human Leukocyte Antigen (HLA) molecules HLA-DQ2 and HLA-DQ8, are necessary but not sufficient to explain the predisposition to CD. Genome wide association (GWA) and follow-up genetic studies have identified several additional genomic regions associated to the disease, which probably contain CD susceptibility genes, but our understanding of the disease pathogenesis is still limited (5).

In fact, the majority of single-nucleotide polymorphisms (SNPs) associated with increased disease risk are located in non-coding regions of the genome (6–8). Some of them map to long non-coding RNAs (lncRNAs) transcripts longer than 200 bases in length, with no protein-coding potential, that have been described to participate in genomic regulation (9). The analysis of the expression profiles of lncRNAs on autoimmune disease-associated regions has shown that they are enriched in autoimmune-disease *loci*, suggesting that they may be crucial to explain GWAS findings (10, 11). Although their mechanisms of action are diverse and still not very well-characterized, lncRNAs regulate gene expression at transcriptional and post-transcriptional levels and are involved in a range of developmental processes, adding complexity to our understanding of how gene expression is regulated (12). On the other hand, expression quantitative trait loci (eQTL) analyses have demonstrated that certain SNPs associated with complex diseases can affect the expression of lncRNAs, and thus lncRNA genes can be considered candidate genes for disease-susceptibility (13). In the case of CD, it has recently been shown that a lncRNA in the *IL18RAP* locus that harbors a disease-associated SNP is a key regulator of genes involved in the NFκB pathway, which is known to be constitutively activated in the small intestine of patients with CD (14, 15).

Apart from GWAS studies, pathway analyses contribute to the reconstruction of altered biological networks that are potentially pathological and could help identify functional candidates that participate to the genetic susceptibility. In the case of CD, although an aberrant immune response is the main force driving the disease, whole genome expression analyses have identified important dysfunctions of other complex biological processes that could be involved in CD development, including alterations in the expression of genes related to intestinal permeability (16–20). Impairment of the epithelial barrier and increased permeability have been shown to be implicated in the development of CD (20) and other gastrointestinal inflammatory diseases like Crohn's Disease (21) and Ulcerative Colitis (22).

The permeability of the intestinal epithelium is dependent on the regulation of intercellular tight junctions (TJ), a continuous, circumferential, belt-like structure at the luminal end of the intercellular space that functions as a barrier. Changes in the expression, distribution, and phosphorylation of TJ proteins have been observed in CD and malfunction of this pathway could have an important role in the augmented intestinal permeability observed in the disease (23, 24). These alterations persist in asymptomatic CD patients who are on GFD (25) and ultrastructural and functional abnormalities in TJs appear also in antibody-negative, asymptomatic first-degree relatives (26), supporting a genetic origin of the pathway alterations and a possible role in the initial stages of the disease. Different studies in European populations have found polymorphisms in several TJ genes, including adapter proteins *MAGI2* and *PARD3*, together with *MYO9B*, a cytoskeleton protein involved in TJ assembly, that are associated with CD (27–29).

The MAGI proteins (Membrane Associated Guanylate-kinase proteins with Inverted domain arrangement) are evolutionarily conserved scaffolding proteins with several protein–protein interaction domains to coordinate signaling complexes (30). MAGI-2 protein (also known as AIP1/S-SCAM/ARIP1) is mainly expressed in the brain at synaptic junctions but has also been identified in the TJ plaque, where it controls epithelial integrity. Interestingly, apart from the association with CD, linkage analyses have shown that *MAGI2* is located in a genomic region harboring susceptibility genes for IBD (31).

With this on mind, we hypothesized that TJ genes could be relevant candidates for gut disorders and specifically for CD. Thus, the aim of the study was to replicate previous association results and to perform gene expression and functional analyses of the potential candidate genes in the associated regions.

## Materials and Methods

### Duodenal Biopsies and Cell Culture

CD was diagnosed at the Pediatric Gastroenterology Clinic (Cruces University Hospital), according to the European Society for Pediatric Gastroenterology, Hepatology, and Nutrition criteria in force at the time of recruitment. The study was approved by the ethics committee of Cruces University Hospital (ref. CEIC-E08/59, CEIC-E13/20, and CEIC-E16/46) and informed consent was obtained from patients or their parents. A total of 48 duodenal biopsy samples were taken from 32 patients divided in 3 categories: 16 biopsies from CD patients at the time of diagnosis (symptomatic and on a gluten-containing diet; 10 girls/6 boys; mean age at diagnosis 2.9 years, range 1.3–9.3 years), 16 biopsies from the same patients after at least 2 years on strict GFD (asymptomatic, antibody negative, and with a recovered intestinal epithelium) and 16 samples from non-celiac individuals (5 girls/11 boys; mean age 7.7 years, range 1.1–13.0 years) who had been subjected to endoscopy but had no signs of gut inflammation. After collection, samples were immediately frozen and stored in liquid nitrogen until use.

The C2BBe1 human colon carcinoma cell line (clone of Caco-2) was selected as a well-established *in vitro* model to study the tight junctions in CD (32, 33). Cells were maintained in DMEM supplemented with 10% heat inactivated FBS, 1% non-essential amino acids and 1% penicillin-streptomycin) in tissue culture flask.

### Association Study

DNA samples from 270 celiac patients and 91 unselected blood donors from the CEGEC (Spanish Consortium for Genetics of Celiac Disease) collection were used for the association study. DNA samples were extracted from whole blood using conventional methods and stored at −80°C until use. The CEGEC study was approved by the Ethics Committees or Institutional Review Board of all participating institutions. Samples were genotyped in the BioMark HD™ platform at the Sequencing and Genotyping Unit of the University of the Basque Country (UPV/EHU). Fluidigm SNP assays were designed according to the protocols recommended by the manufacturer (Fluidigm Corporation, San Francisco, CA, USA) (Table S1). We selected 3 SNPs in the *MAGI2 locus*, 2 in *PARD3*, and 3 in *MYO9B*, based on previously published association results (29, 34, 35). The SNP Genotyping Analysis software by Fluidigm was used for genotype calling. Clusters were evaluated manually, and doubtful calls were recalled or rejected. To ensure population homogeneity, an identity-by-state analysis and multidimensional scaling were performed before standard χ2 association analysis using Plink 1.07 (36).

### Analysis of Coding Potential of RP4-587D13.2

The protein-coding capacity of *RP4-587D13.2* was evaluated *in silico*. All possible open reading frames (ORFs) were identified using ORF Finder (NCBI) (37) and the coding potential of the transcript was assayed with Phylogenetic Codon Substitution Frequencies (PhyloCSF) based on phylogenetic conservation (38), as well as with the coding potential assessment tool (CPAT) that uses a model built with open reading frame size and coverage together with codon and hexamer usage bias (39).

### Cellular Fractionation

To analyze the distribution of *RP4-587D13.2* RNA in the nuclear and cytoplasmic cell compartments of C2BBe1 cells, nuclei were isolated using hypotonic lysis buffer (10 mM Hepes, 10 mM KCl, 0.1 mM EDTA and %0.8 Triton-X) as previously described. For relative quantification, the amounts of nuclear *RP4-587D13.2, Lnc13* (nuclear control) and *RPLP0* (cytoplasmic control) RNA were measured by RT-QPCR and compared to the amount of each RNA in whole cells.

### Cell Dicer-Substrate Short Interfering RNAs (DsiRNAs) Transfection and Gliadin Exposure

For *in vitro* cell transfection and gliadin stimulation, 5 × 10^4^ cells/well were seeded in 24-well plates with 500 μl of supplemented medium without antibiotics. Reverse transfection was performed with 30 nM of DsiRNA against human *MAGI2* (IDT corp. Cat. No. hs.Ri.MAGI2.13.1), *RP4-587D13.2* (5′ -GCAGUUUAAUAUCAUGUAUUGAAAA-3′) or negative control DsiRNA (IDT corp. Cat. No. 51-01-14-01) using Lipofectamine RNAimax reagent (Thermo Fisher Scientific, Waltham, MA, USA) and following the manufacturer's instructions. Samples were incubated overnight in 5% CO_2_ at 37°C before adding fresh medium with antibiotic, then incubated again for a total of 48 h from the start of transfection. Then a well-established protocol was used to mimic gliadin-induced inflammatory response *in vitro* (40–42). Cells were exposed to 1 mg/ml of pepsin-trypsin digested gliadin (PTG) or BSA (PT-BSA) for 4 h and harvested for RNA or protein extraction. Pepsin-trypsin digest of gliadin (PTG) (Merck, Darmstadt, Germany 1.07185; 9002-07-7, and G3375-25, respectively) was prepared as described previously (43). An enzymatic digest of BSA (PT-BSA) was prepared in the same way and used as a negative control of stimulation.

### Protein Extraction and Immunoblot Analysis

Whole cell extracts were directly lysed into 2x Laemmli Sample buffer containing 5% β- mercaptoethanol and stored at −20°C until use. Total protein extracts were heat-treated at 95°C for 5 min, fractionated by SDS-PAGE and transferred to nitrocellulose membranes using the Trans-Blot Turbo Transfer apparatus (BioRad, Hercules, California, USA). After incubation with 5% non-fat milk in Tris-buffered saline with 0.05% Tween (TBS-T) for 1 h, the membrane was incubated at 4°C overnight with polyclonal antibodies against MAGI2 (1:500) (ABCAM, Cat. No. ab97343) and β-Tubulin (1:1,000) (ABCAM, Cat. No. ab18207). Membranes were washed three times for 10 min and incubated with horseradish peroxidase-conjugated anti-mouse (1:2,000) or anti-rabbit (1:10,000) antibodies for 1 h at room temperature in 5% BSA in TBS-T (Jackson ImmunoResearch Laboratories, Inc.; Cat No. 111-035-003 and 115-035-003, respectively). Blots were washed with TBS-T three times and visualized using SuperSignal West Femto Maximum Sensitivity Substrate (Thermo Fisher, Cat No. 34094) on the ChemiDoc MP system (BioRad, Hercules, California, USA). The quantification of band intensities was done using the Image Lab 4.1 software and results were normalized to β-tubulin.

### RNA and DNA Isolation From Tissue Samples and Cultured Cells

Total RNA and DNA were prepared with the NucleoSpin RNA kit and NucleoSpin Blood kit, respectively (Macherey-Nagel, Düren, Germany) according to the protocols provided. Frozen biopsy samples were disrupted with disposable plastic pellet pestles prior to nucleic acid extraction. Cells were lysed and homogenized using the buffers supplied with the kits. The concentration and purity of the RNA and DNA were determined by UV absorbance at 260 nm on a NanoDrop 1000 spectrophotometer (Thermo Fisher Scientific, Waltham, MA, USA) and samples were stored at −80°C until use. To determine the expression of *MAGI2* and *RP4-587D13.2* across different human tissues, a commercial Human Total RNA Master Panel II (Clontech Laboratories, Inc., Cat. No. 636643) was used.

### Expression Analyses

The expression of *MAGI2* and *RP4-587D13.2* was analyzed in biopsy samples and caco2 cells. Total RNA was reverse transcribed with the iScript cDNA Synthesis Kit (Bio-Rad Laboratories, Inc. Berkeley, CA, USA). Expression of *MAGI2* mRNA was determined by real-time (RT)-PCR using specific TaqMan Gene Expression Assays (thermoFisher Scientific) in a Eco Real-Time PCR System (Illumina, Inc., San Diego, CA, USA). The expression of *RP4-587D13.2* lncRNA was measured using custom-designed primers (F: 5′-GGTGCTGGAAATTCATCAGTG-3′; R: 5′-TGACCACATGACTGACACCA-3′) and Quantitect SYBR Green master mix (Qiagen, Hilden, Germany). The specificity of the primers was tested by *in-silico* PCR on the University of California Santa Cruz web server (http://genome.ucsc.edu/cgi-bin/hgPcr) and melting curve analysis were performed in all qPCR reactions.

The expression levels of TJ-related genes were measured by RT-PCR on a Fluidigm BioMark dynamic array system following the 48.48 Fast Real-Time PCR Workflow (Fluidigm Corporation, San Francisco, CA, USA). Candidate genes with central functions in the TJ and TJ-related Toll Like Receptor (TLR) networks according to the KEGG database (http://www.genome.jp/kegg) were selected (Table S2). The reverse transcribed cDNA from each sample was preamplified using the Fluidigm Gene Expression Specific Target Amplification kit (Fluidigm Corporation, San Francisco, CA, USA) and amplified using TaqMan Universal PCR master mix (Thermo Fisher Scientific, Waltham, MA, USA). Complete results are available at the Gene Expression Omnibus (https://www.ncbi.nlm.nih.gov/geo/) with accession number GSE84729. The expression of the housekeeping gene *RPLP0* (large ribosomal protein) was quantified in each experiment and used as an endogenous control of input RNA. The relative expression of each gene was calculated using the accurate Ct method as previously described (44).

The Commercial TaqMan assays probes (Thermo Fisher Scientific, Waltham, MA, USA) used for expression analyses are listed in Table S2. Probes that spanned an exon–exon junction were chosen to avoid the detection of genomic DNA.

### SNP Genotyping

In order to determine whether the risk attributed to the newly identified variant rs6962966 was related to gene expression in CD patients, the tag SNP rs2691527 (*r*^2^ = 1 with associated SNP rs6962966) was genotyped in the duodenal biopsy samples on a Eco Real-Time PCR System (Illumina, Inc., San Diego, CA, USA) using a predesigned TaqMan Genotyping assay (Thermo Fisher Scientific, Assay ID 4351379) and TaqMan genotyping Mastermix (Thermo Fisher Scientific, Waltham, MA, USA).

### Analysis of Results

Statistical analyses were carried out using GraphPad Prism version 5.0 software (GraphPad Software Inc, La Jolla, CA, USA) to assess differences among groups. Non-parametric, paired (Wilcoxon matched-pairs test) tests were performed for the comparison of the expression levels between CD patients at diagnosis and after 2 years on GFD, and the Mann–Whitney U test was used to compare each disease group to the controls and unpaired t test to in the silencing experiments. Coexpression was assessed with Spearman's correlation test. Heat-maps were constructed using the FiRe Macro v2.2 for Microsoft Excel from the University of Fribourg available at http://www.unifr.ch/plantbio/FiRe/main.html. Only 2-tailed *p*-values below 0.05 were considered significant.

## Results

### An Intronic Polymorphism in *MAGI2* Is Associated to CD

We analyzed 8 SNPs located in *MAGI2, PARD3*, and *MYO9B*, previously associated with CD. Their frequencies in CD patients and controls are listed on Table 1. We detected an association between SNP rs6962966 (GRCh38/hg38; chr7:77,804,123) in an intronic region of *MAGI2* and CD risk. The SNP was in Hardy-Weinberg equilibrium in the control group and the frequency of the minor allele was higher in patients, augmenting the risk to develop CD 1.88-fold (95% CI = 1.24–2.87; *p* = 0.0029). No other SNPs in *MAGI2, PARD3*, or *MYO9B* were associated with CD.

**Table 1:** Association analysis of single nucleotide polymorphisms in TJ-related candidate genes in CD.

**SNP**	**Position (Hg38)**	**Gene**	**Minor allele**	**CD MAF**	**Control MAF**	***p*-value**	**Odds-ratio (CI 95%)**
**rs6962966**	**chr7: 78174806**	***MAGI2***	**A**	**0.5210**	**0.3661**	**0.0029**	**1.88 (1.24–2.87)**
rs9640699	chr7: 78366115	*MAGI2*	A	0.3613	0.3056	0.1763	1.29 (0.89–1.85)
rs1496770	chr7: 78629694	*MAGI2*	T	0.4699	0.4341	0.4022	1.16 (0.82–1.62)
rs10763976	chr10: 34275364	*PARD3*	G	0.4713	0.4560	0.7246	1.06 (0.76–1.49)
rs4379776	chr10: 34328092	*PARD3*	T	0.3811	0.4286	0.2583	0.82 (0.58–1.16)
rs2305767	chr19: 17183487	*MYO9B*	C	0.3547	0.3956	0.3311	0.85 (0.59–1.19)
rs1457092	chr19: 17193427	*MYO9B*	A	0.3940	0.3889	0.9042	1.02 (0.72–1.45)
rs2305764	chr19: 17203024	*MYO9B*	A	0.4147	0.4333	0.6710	0.93 (0.65–1.32)

*Significant association values (p < 0.05) are shown in bold*.

*Chr, chromosome; SNP, single nucleotide polymorphism; MAF, minor allele frequency; CD, Celiac disease; OR, odds ratio; CI, confidence interval*.

**Figure 1:**
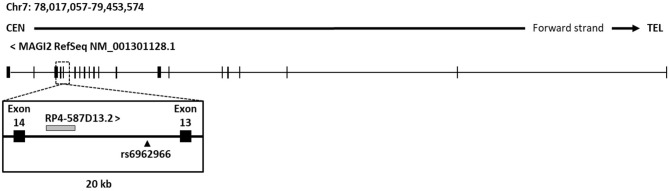
Genomic context of CD associated SNP rs6969266. The reference sequence of *MAGI2* (NCBI) and genomic annotations of a 20-kb region around candidate SNP rs6962966 are shown.

In order to characterize the effects of the associated intronic SNP on disease risk, we analyzed its genomic context in search for putative regulatory elements that could be affected causing alterations in gene regulation. An analysis of current UCSC Genome browser annotations showed that protein-coding gene *MAGI2* hosts different types of non-coding genes, including processed transcripts, pseudogenes, and RNA genes. The great majority of them are located in introns and no specific function or effect on their host gene have been described so far. As an exception, the antisense gene *MAGI2-AS3* overlaps the first exon of *MAGI2* and has been recently linked to breast cancer (45). Regarding the intron where the CD-associated rs6962966 SNP falls, we found that it also harbors a non-characterized transcript, named *RP4-587D13.2* (Figure 1).

### *RP4-587D13.2* Is a Long Non-coding Transcript That Can Regulate *MAGI2* Expression

**Figure 2:**
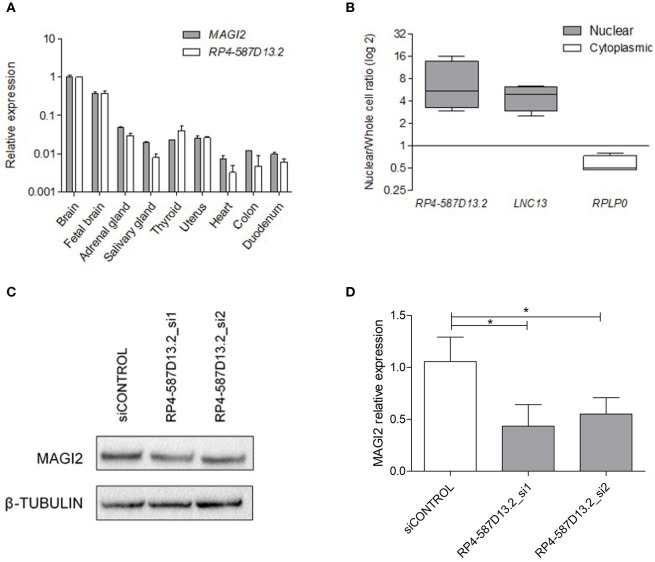
Characterization of *RPL4-587D13.2* expression in human tissues and the C2BBe1 cell line. **(A)** mRNA expression of *MAGI2* and *RPL4-587D13.2* in RNA from 9 human tissues. Values are represented relative to the tissue with the highest expression levels (brain). **(B)** Subcellular localization of *RPL4-587D13.2* in C2BBe1 cells. The box-plots show the ratio of nuclear to whole cell transcripts, represented as the maximum and minimum values and the mean (*n* = 3); *LNC13* and *RPLP0* are used as nuclear and cytoplasmic controls, respectively. **(C)** Representative western blot and **(D)** quantification of MAGI2 protein levels in C2BBe1 cells silenced for *RPL4-587D13.2* (siRNA_1 and siRNA_2). The mean and standard deviation of *MAGI2* expression relative to the control siRNA are shown (*n* = 3) (*0.01 < *p* < 0.05).

In order to characterize the region around the associated SNP, we performed expression analysis of both *RP4-587D13.2* and *MAGI2* across different human tissues. The results showed that these transcripts are ubiquotiusly expressed in all human tissues analyzed, including the intestine, the CD target tissue, and are predominantly expressed in brain (Figure 2A). It can be observed that both present a similar expression pattern and are positively correlated (*p* = 0.0007) across tissues. We next evaluated the protein-coding capacity of *RP4-587D13.2 in silico* using three different approaches. Overall, the lack of functional ORFs as well as the low phylogenetic conservation and coding probability of the region suggests that *RP4-587D13.2* is a long non-coding transcript (Table S3).

The cellular localization of the lncRNA was evaluated in C2BBe1 intestinal human cells, and results revealed that *RP4-587D13.2* accumulates preferentially in the nucleus (Figure 2B). The nuclear localization of *RP4-587D13.2* together with its coexpression with the neighboring *MAGI2* suggests that this lncRNA could contribute to the regulation of *MAGI2* expression. To test this hypothesis, we performed siRNA silencing *of RP4-587D13.2* in C2Bbbe1 intestinal cells using two different siRNAs. We managed to reduce the expression of RP4-587D13.2 by 46% in intestinal cells (Figure S1) which in turn resulted in a 56% decrease in MAGI2 protein (Figures 2C,D), confirming that this lncRNA is somehow affecting the regulation of the coding gene.

### PTG Stimulation Alters the Expression of TJ-Related Genes in *MAGI2*-Silenced Cells

**Figure 3:**
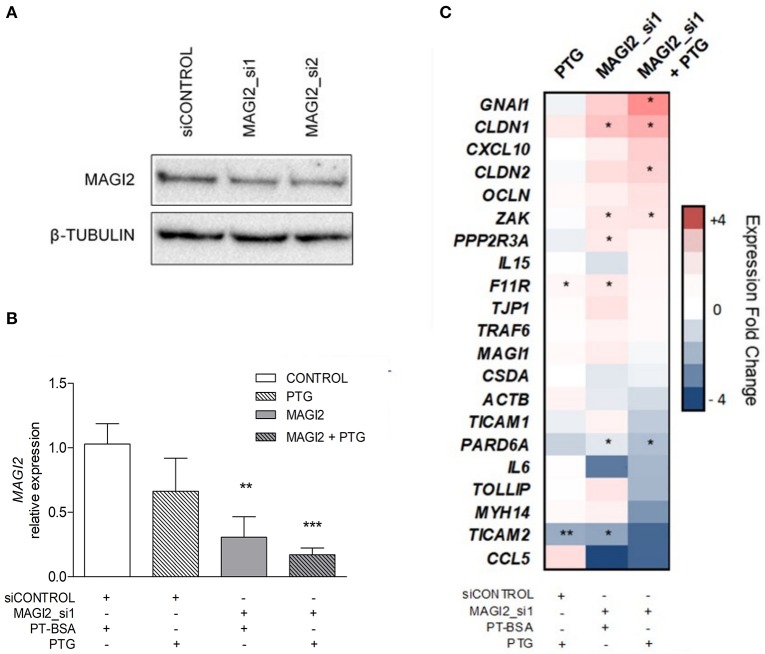
Effects of *MAGI2* silencing and gliadin on the expression of a TJ-related genes in C2BBe1 intestinal cells. **(A)** Western blot of MAGI2 protein upon silencing in C2BBe1 cells. **(B)** Quantification of MAGI2 protein levels under different cell treatments, relative to control cells (transfected with control siRNA and incubated with PT-BSA). Mean and standard deviation are shown (*n* = 3). **(C)** Heat map of candidate gene expression in different conditions; PTG stimulation, siRNA mediated MAGI2 silencing (MAGI2_si1) and PTG stimulation of MAGI2_si1 cells. Expression levels are shown as fold-change relative to the average of control samples (silenced with a control siRNA and incubated with PT-BSA). Columns represent treatment categories and lines candidate genes (*0.01 < *p* < 0.05; **0.001 < *p* < 0.01; ***p < 0.001).

In order to determine the functional significance of the *MAGI2* region in the CD characteristic dysregulation of the TJ pathway, the expression of a TJ-related gene panel was analyzed in the C2BBe1 intestinal cell line. Due to the difficulty of efficiently silencing the lncRNA, probably because of its nuclear location, the effects in downstream gene expression were assessed by *MAGI2* silencing. As expected, the efficiency of *MAGI2* silencing was above 59% (Figures 3A,B) and silenced cells showed altered expression of *CLDN1, F11R, PARD6A, PPP2R3A, TICAM2*, and *ZAK* (Figure 3C). Interestingly, upon PTG stimulation, there was an additional decrease of *MAGI2* and a stronger effect on the TJ-related genes in *MAGI2*-silenced cells: the changes observed in *PARD6A* and *ZAK* were more pronounced, and alterations extended to other genes of the pathway, including *GNAI1* and *CLDN2*.

### The Expression of *MAGI2* and *RP4-587D13.2* Is Reduced in CD and Could Explain Alterations of Other Tight Junction Genes

**Figure 4:**
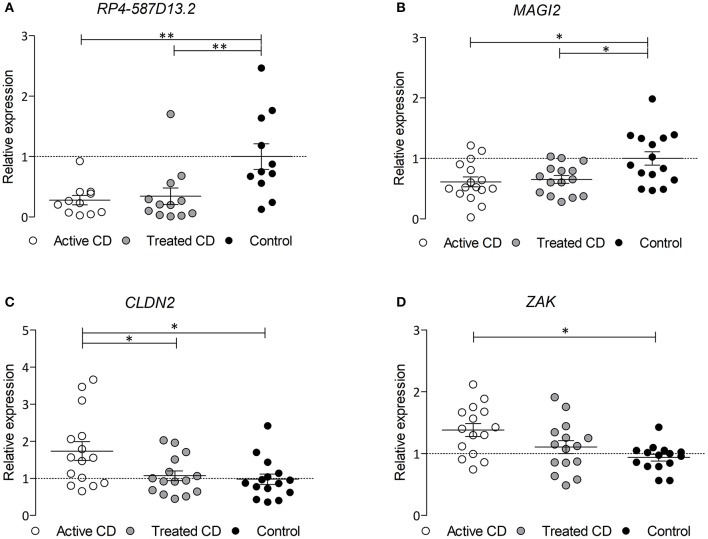
mRNA expression of **(A)** *RPL4-587D13.2*, **(B)** *MAGI2*, **(C)** *CLDN2*, and **(D)** *ZAK* in duodenal biopsies from CD patients at diagnosis (white dots), treated CD patients (gray dots) and non-Celiac controls (black dots) represented as mean and SEM, relative to the average of the control samples (*0.01 < *p* < 0.05; **0.001 < *p* < 0.01).

Since the region harboring *RP4-587D13.2* and *MAGI2* is associated to CD and we have observed that both genes can influence the expression of other TJ-related genes, we analyzed their expression in biopsies from CD patients and controls. As shown in Figures 4A,B, both *MAGI2* and *RP4-587D13.2* show significantly lower expression levels in the duodenum of active CD patients, and this downregulation persisted after GFD treatment. The correlation between *MAGI2* and *RP4-587D13.2* gene expression previously observed in different human tissues (Figure 2A) was also present in duodenal biopsies (*r* = 0.4033; *p* = 0.027) (Figure S2).

In order to assess whether the TJ genes that are altered by the downregulation of the *MAGI2* region are also altered in CD, we analyzed their expression in CD and control biopsies. Two of the genes, *CLDN2* and *ZAK*, are overexpressed in the intestine of active CD patients (on a gluten containing diet) compared to treated and control samples (Figures 4C,D), confirming what had been previously observed *in vitro* (Figure 3C).

**Figure 5:**
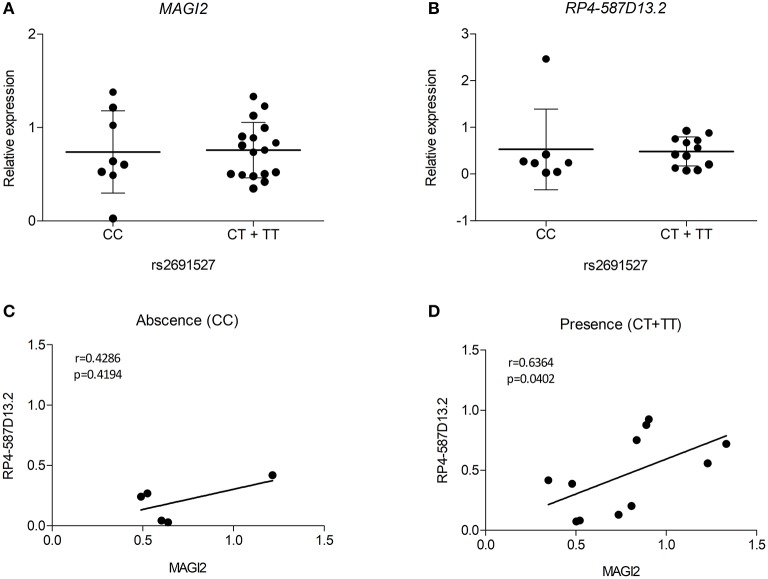
eQTL analyses for **(A)** MAGI2 and **(B)** RP4-587D13.2 in duodenal biopsies from patients with CD and non-CD controls. Scatter plots show expression relative to the control mean in patients lacking or harboring the tag SNP rs2691527*T allele. Spearman correlation between MAGI2 and RP4-587D13.2 expression levels according to the **(C)** absence or **(D)** presence of tag SNP rs2691527*T allele.

Despite the limited number of biological samples representing all three genotypes in our study, we also searched for relationships between the risk genotypes and the expression level of the *MAGI2* region genes. As the presence of all three genotypes in every group was not possible, heterozygous, and risk-allele homozygous samples were combined. The results of eQTL analysis showed that the presence of the risk allele was not significantly associated with decreased expression of either transcript in the *MAGI2* region (Figures 5A,B). However, when we analyzed the correlation of both transcripts based on the genotype we observed that the samples harboring the risk allele showed a stronger correlation (*r* = 0.6364; *p* = 0.0402) than those with no risk allele (*r* = 0.4286; *p* = 0.4194) (Figures 5C,D) suggesting that the risk allele strengthens the interaction of both genes thus affecting the regulation of downstream genes.

## Discussion

Polymorphisms in TJ-related genes *MAGI2, PARD3*, and *MYO9B* that potentially influence the homeostasis of the intestinal barrier have been associated with risk of gastrointestinal disorders like CD and IBD (27, 29, 46). However, attempts to replicate those associations in different cohorts have sometimes failed, highlighting the heterogeneity and complexity of common human disorders (34, 35, 47). In this study, we have been able to replicate the association with rs6962966 (an intronic variant in *MAGI2*), previously identified in Dutch and British cohorts (29), in a Spanish CD cohort.

Human *MAGI2* is a relatively large gene (~1.5 Mbps) that maps to chromosome 7q21 and encodes the membrane-associated, guanylate kinase inverted 2 tight junction protein, involved in epithelial integrity. The CD-associated allele has been proposed to be involved in *MAGI2* gene function, but no studies have been performed to determine the possible effects of the CD-associated polymorphism. The candidate gene selection that follows SNP association studies is often aprioristic, and functional studies are the only unbiased approach to characterize the associated region. Therefore, in order to define the role of this intronic variant in CD pathogenesis, we scrutinized the genomic region around rs6962966 and found that, apart from the coding gene *MAGI2*, the region also harbors *RP4-587D13.2*, a long RNA transcript represented by a cluster of expressed sequence tags (ESTs) covering 721 bp that maps 3,9 kb upstream (centromeric) of the CD-associated SNP. *In silico* analysis showed that this transcript is a long non-coding intronic RNA of unknown biological function. Recent studies have evidenced that intronic regions are key sources of regulatory non-coding RNAs that are able to regulate the expression of other genes through diverse mechanisms (48). Interestingly, the antisense lncRNA *MAGI2-AS3* overlapping the first exon of *MAGI2* has been recently related to tumor suppression in several cancers including breast, lung and hepatocellular carcinoma (45, 49, 50). In the case of lncRNA *RP4-587D13.2*, located within the intron harboring the CD-associated rs6962966, both its differential tissue distribution and preferential nuclear localization suggest that it is a functional, regulatory transcript. Interestingly, *RP4-587D13.2* and *MAGI2* show a concordant expression profile across human tissues, including the small intestine, indicating that *RP4-587D13.2* could be processed from the same pre-mRNA and could be functionally related to *MAGI2*. Moreover, *MAGI2* shows decreased protein levels upon silencing of this intronic lncRNA, suggesting that *RP4-587D13.2* could be involved in the transcriptional (or post-transcriptional) regulation of *MAGI2* expression and thus in the coordination of the expression of genes that are relevant to the TJ pathway. However, these regulatory relationships between the intronic lncRNA and the protein transcribed by its host *locus MAGI2* must be confirmed and the mechanisms that underlie such regulatory behaviors remain to be fully understood.

We observed that the expression of several TJ-related genes is altered in an *in vitro* model that mimics the context of CD, where the expression of *MAGI2* is repressed and intestinal cells are exposed to digested gliadin (PTG), suggesting that both *MAGI2*, and the upstream *RP4-587D13.2* are necessary for a correct function of the pathway. Moreover, the decrease in expression of both genes, together with the alteration observed in several other TJ genes in the disease tissue suggests that the *MAGI2* region could be involved in the regulation of the network. Interestingly, in the *in vitro* model, gliadin stimulation enhanced the effects of gene-silencing, resembling what is observed for *CLDN2* and *ZAK* in biopsy samples from active and treated CD patients. On the other hand, the expression of *MAGI2* and *RP4-587D13.2* is downregulated in the duodenum of active CD patients and persists even after more than 2 years on GFD and an apparent recovery from intestinal atrophy, supporting a constitutive alteration that could have a genetic origin. However, several other TJ genes that are altered in the PTG-challenged *in vitro* model (and in active CD) are normalized in GFD-treated patients, stressing the role of gluten-mediated induction of some of the alterations. The constitutive alteration of gene expression in the *MAGI2* region could promote an increased response to ingested gluten, probably leading to a higher disruption of TJ structures and to an enhanced intestinal permeability. Overall, the constitutive alteration of gene expression, together with the presence of an associated genetic variant point to a dysfunction that is present before the clinical onset of the disease, as has been previously suggested for the enhanced intestinal permeability that occurs in CD (25).

Several studies have described that more than half of the disease-associated SNPs correlate with the expression of at least one adjacent gene (51) acting as *cis-eQTL*. The eQTL analysis in our samples did not find a link between the risk allele and the reduction of *MAGI2* or *RP4-587D13.2* expression in intestinal biopsy samples. However, there was a stronger correlation between the expression of both genes in the presence of the risk allele, suggesting that the associated SNP could be influencing the regulation of *MAGI2* exerted by *RP4-587D13.2*. The effects of disease-associated SNPs go far beyond the simplistic idea of transcriptional control of an adjacent *locus* and the complexity of the mechanisms involved in the functional translation of associated genetic variants make it difficult to explain their role in the disease. Further functional studies and large meta-analyses might be able to pinpoint the causal variants within a disease-associated locus and determine their downstream effects in greater detail (52).

In summary, we have demonstrated that the *MAGI2* genomic region is associated with CD and presents transcriptional alterations that seem to be relevant for disease pathogenesis. We postulate that RP4-587D13.2 is regulating *MAGI2* expression, that this relationship seems to be affected by the associated SNP and that the constitutive reduced expression of both transcripts in patients could affect the TJ pathway, which is crucial for intestinal permeability, and enhance the response to the ingested gluten. Further studies are needed to verify the functionality of this intronic lncRNA and the complex regulatory relationships between RP4-587D13.2, its host loci *MAGI2* and disease. All this will help to understand the complexity of the interactions between the environmental trigger, genetic polymorphisms and gene expression.

## Data Availability Statement

The datasets generated for this study can be found in the Gene Expression Omnibus GSE84729.

## Ethics Statement

The studies involving human participants were reviewed and approved by Ethics Committee of Cruces University Hospital (ref. CEIC-E08/59, CEIC-E13/20 and CEICE16/46). Written informed consent to participate in this study was provided by the participants' legal guardian/next of kin.

## Author Contributions

AJ-M designed and performed experiments, analyzed and interpreted data, and wrote the manuscript. IS contributed with the experimental design and procedures and data analysis. KG-E analyzed and interpreted data. AO-G performed experiments and analyzed data. IR-G and MS performed experiments and collected samples. II performed sample collection. AC-R conceived the ideas of experimental design of the study, performed experiments, analyzed and interpreted data, and provided revisions to scientific and grammatical content of the manuscript. JB conceived the ideas of experimental design of the study, interpreted data, provided revisions to scientific, and grammatical content of the manuscript and provided funding.

### Conflict of Interest

The authors declare that the research was conducted in the absence of any commercial or financial relationships that could be construed as a potential conflict of interest.

## Abbreviations

BSA: Bovine serum albumin
CD: Celiac disease
CEGEC: Spanish Consortium for the Genetics of Celiac Disease
CPAT: Coding potential assessment tool
DsiRNA: Dicer-substrate short interfering RNAs
EST: expressed sequence tag
eQTL: Expression quantitative trait loci
GFD: Gluten-free diet
GWA: Genome wide association
HLA: Human Leukocyte Antigen
IBD: Inflammatory bowel disease
lncRNA: Long non-coding RNA
OR: Odds ratio
ORF: Open reading frame
PhyloCSF: Phylogenetic Codon Substitution Frequencies
PT-BSA: Pepisn-trypsin digested BSA
PTG: Pepsin-trypsin digested gliadin
RT-qPCR: Quantitative reverse transcription PCR
si-RNA: Small interfering RNA
SNP: Single-nucleotide polymorphism
TBS-T: Tris-buffered saline with Tween
TJ: Tight Junction
TLR: Toll-like receptor
WB: Western blot.
