# Supplementary material for: MAGI2 Gene Region and Celiac Disease
Source: Front Nutr. 2019 Dec 19;6:187. doi: 10.3389/fnut.2019.00187 (PMC6930898; doi:10.3389/fnut.2019.00187)
Supplement: Supplementary file 1 [file Data_Sheet_1.ZIP › Supplementary material/Supplementary_Material.docx]

# Supplementary Figures and Tables

Table S1. Primers for the SNP genotyping experiment. ASP1 = SNP allele detected with allele-specific primer 1, ASP2 = SNP allele detected with allele-specific primer 2, SNP_SEQ = sequence of the amplified fragment containing the SNP, ASP1_SEQ = sequence of allele-specific primer 1, ASP2_SEQ = sequence of allele-specific primer 2, LSP_SEQ = sequence of locus-specific reverse primer, STA_SEQ = sequence of forward primer for specific target amplification.

Table S2. Candidate genes and Taqman assays used for expression analyses.

Table S3. In silico analysis of the coding potential of RP4-587D13.2. Results of ORF Finder, PhyloCSF and CPAT tools search are shown. ORFs shorter than 100 aa are not consider to have a coding probability. Negative score of the PhyloCSF is likely to represent a conserved noncoding sequence. Codon Ficket score, and hexamer score usage bias with CPAT results in the coding probability value. Values lower than 0.364 are considered noncoding transcripts.

Figure S1. RP4-587D13.2 lncRNA silencing in C2BBe1 intestinal cell line. Relative expression to control (cells transfected with siRNA control) is shown. Mean and standard error of the mean are represented (n=4).

Figure S2. Spearman correlation between MAGI2 and RPL4-587D13.2 gene expression levels.

| **ASSAY_ID** | **SNP_ NAME** | **ASP1 / ASP2** | **SNP_SEQ** | **ASP1_SEQ** | **ASP2_SEQ** | **LSP_SEQ** | **STA_SEQ** |
| --- | --- | --- | --- | --- | --- | --- | --- |
| GTA0093555 | rs6962966 | A / G | TCACCAATAATCTTAGGGTAGGGGCTGACCATACCCGAAAGACCAGCATTGTAATTAGAGGGTTGTGTCTTTGAGCCACATGATATCAGCCTGGCCTCTG[A/G]GGAAGAAAGGGGGCTGAAGATTGAGGTCATCCTTGTGGGCAATGGTTCAATCAATCATGCCCATGTAACAAAACACTGG | GATATCAGCCTGGCCTCTGA | GATATCAGCCTGGCCTCTGG | TGCCCACAAGGATGACCTCA | GGTTGTGTCTTTGAGCCACAT |
| GTA0093556 | rs9640699 | A / C | CGTAAGCTTTTTTTGCTACTGGRTTGGAAGGTGGAMTCTTTTTCATAYCTTGTAAAGAGTACCTCCAATTGTATGAATGSGTGTGTAATTAAACATAAGA[A/C]TGATAGCTGTACAGAGAATCCACTGCTYATTATCACTAATGGCATTAGTAAATCAACATTTTCTTGACTATATATTAAC | CAGTGGATTCTCTGTACAGCTATCAT | CAGTGGATTCTCTGTACAGCTATCAG | CTTGTAAAGAGTACCTCCAATTGTATGAATG | GCAGTGGATTCTCTGTACAGC |
| GTA0093558 | rs1496770 | C / T | TTTCATTACTGACAGCAGTTGCTATAAAAAAACAAAAACTTTCTCTGACAAAGTACTCMACAAATTCAGATTACTTAATGTCAGTTTGATCCTCCTGATG[C/T]TTGGTAATTCTTTTATGTGTGTCTTCTTGTCTCTCTCTCTTATATTCCCAACATTTTCTATATTCAACTGCTTTGACGT | TGTCAGTTTGATCCTCCTGATGC | AATGTCAGTTTGATCCTCCTGATGT | AGAGAGAGACAAGAAGACACACATAAAAGAATT | ACAAATTCAGATTACTTAATGTCAGTTTGA |
| GTA0093550 | rs10763976 | G / A | ACCTGCATCACTGCTTTCCAATGTGGCAGCTTACACTGGTGGTGAAAACCTTAGAATAAAACTATAGCATACGTGTGGTCCACCAGGTAGAACTACTGCC[G/A]CAAAAGGCTAACGGATCTGGTCAGTAGCCTGAACTAATGTGYATTCTCAAGTTCATTCTAGAAATTAATGCYAACGAGG | CCAGATCCGTTAGCCTTTTGC | ACCAGATCCGTTAGCCTTTTGT | CGTGTGGTCCACCAGGTAGA | ACATTAGTTCAGGCTACTGACCA |
| GTA0093551 | rs4379776 | C / T | CAGGTCCACAAGCCAGACGCTGAAAWTGGTGTGGTTAGGAAGCCRAAGGAGGGCTAACTGAGCCAGCCKTCTTTCCAAAGCTGGAGGCAGAAAGTGCCTT[C/T]GCGTCTTCAGTTTTATGTAAGCTGAYAGCCRCCAGCAGTAACACCACATTTGATAGACAAWTTTTTTTTAAACAAAAAC | CAGCTTACATAAAACTGAAGACGCG | CAGCTTACATAAAACTGAAGACGCA | TCTTTCCAAAGCTGGAGGCAGA | TCAAATGTGGTGTTACTGCTGG |
| GTA0093559 | rs2305767 | C / T | GGTCACCTGGTCACCACCAGGACTGCTCAGGCTCCCTCCGCCCCACTSGGCATCCTGCTGTGATCTGGGGGATATGTCAGTTCCTCCATAGCAAGCCCCG[C/T]TGGATGCACGTCCCACCCTGTAGATTCCTAGCCAGCCCCTCCTACTTCTCAGACATCSGAATGGYCCCCAGACTAAACA | GGTGGGACGTGCATCCAG | GGTGGGACGTGCATCCAA | GCATCCTGCTGTGATCTGGG | GGGCTGGCTAGGAATCTACA |
| GTA0093552 | rs1457092 | C / A | GGATCGGTCAGGGAACACCTGGATTCAGGGTTGGAGGGGCTGCCTGGACCAGCCCARCACTCACATGGAGCTGGGGCATCCACCGGGCACAGAGAAGCCC[C/A]CAGGAGGATATCAGCAGCTCCCGTCCCAGCACCCACAGCTCCGAGCCCAGCCCCTCCCCGACCTAGGCACCCTGTTCAC | GAGCTGCTGATATCCTCCTGG | GAGCTGCTGATATCCTCCTGT | CATCCACCGGGCACAGAGA | CTGGGCTCGGAGCTGT |
| GTA0093554 | rs2305764 | G / A | CATTGGCAGGAGCATGCAYGTGTGTGTCAGTGTCAATGTGCGCATGGGCCCRTCTGCACGCGTATGTGTGCRTGGACAYGTGTGAGTGTGTTTTTCCCCC[G/A]GCATATACGGAGCCGTAGTCTTGAATAAGTCACCCCTGAGATCCCATGGGTCGTYATCCACCAGTGGCTGGGGAGGGCT | GTGAGTGTGTTTTTCCCCCG | TGTGAGTGTGTTTTTCCCCCA | ACGACCCATGGGATCTCAG | TCTGCACGCGTATGTGT |

| **Gene symbol** | | **Assay code** | **Encoding protein** | **Protein family** |
| --- | --- | --- | --- | --- |
| **1** | *CLDN1* | Hs00221623_m1 | Claudin 1 | **TJ Transmembrane proteins** |
| **2** | *CLDN2* | Hs00252666_s1 | Claudin 2 |  |
| **3** | *F11R* | Hs00170991_m1 | Junctional adhesion molecule 1 (JAM-1) |  |
| **4** | *OCLN* | Hs00170162_m1 | Occludin |  |
| **5** | *MAGI1* | Hs00191026_m1 | Membrane associated guanylate kinase 1 | **TJ Adaptor Proteins** |
| **6** | *MAGI2* | Hs00202321_m1 | Membrane associated guanylate kinase 2 |  |
| **7** | *PARD6A* | Hs00180947_m1 | Partitioning defective 6 homolog α (C. elegans) |  |
| **8** | *TJP1* | Hs01551861_m1 | Zonula occludens 1 (ZO-1) |  |
| **9** | *YBX3* | Hs01124964_m1 | ZO-1-Associated Nucleic Acid-Binding (ZONAB) | **TJ Regulators** |
| **10** | *GNAI1* | Hs01053353_m1 | G protein, alpha inhibiting activity polypeptide 1 |  |
| **11** | *PPP2R3A* | Hs01097014_m1 | Protein phosphatase 2, regulatory subunit B,α |  |
| **12** | *ZAK* | Hs00370447_m1 | Sterile α motif & leucine zipper containing kinase |  |
| **13** | *ACTB* | Hs01060665_g1 | Actin, beta | **Cytoskeletal Filaments** |
| **14** | *MYH14* | Hs00226855_m1 | Myosin, heavy chain 14, non-muscle |  |
| **15** | TICAM1 | Hs00706140-s1 | Toll Like Receptor Adaptor Molecule 1 | **TLR related** |
| **16** | TICAM2 | Hs04189225_m1 | Toll Like Receptor Adaptor Molecule 2 |  |
| **17** | TOLLIP | Hs01553188_m1 | Toll Interacting Protein |  |
| **18** | TRAF6 | Hs00371512-g1 | TNF Receptor Associated Factor 6 |  |
| **19** | CCL5 | Hs00982282_m1 | C-C Motif Chemokine Ligand 5 | **Cytokines** |
| **20** | CXCL10 | Hs01124251-g1 | C-X-C Motif Chemokine Ligand 10 |  |
| **21** | IL15 | Hs01003716_m1 | Interleukin 15 |  |
| **22** | IL6 | Hs00174131_m1 | Interleukin 6 |  |
| **23** | *RPLP0* | 4333761F | Large ribosomal protein, Endogenous Control |  |

|  |  | **ORF Finder** | | | |  | **PhyloCSF** |  | **CPAT** | | | |
| --- | --- | --- | --- | --- | --- | --- | --- | --- | --- | --- | --- | --- |
| **Data ID** | **RNA size** | **ORF size** | | **Strand & frame** | **Start / Stop** |  | **PhyloCSF score** |  | **Ficket score** | **Hexamer score** | **Coding probability** | **Coding label** |
| **RP4-587D13.2** | **721 bp** | 1 | 171 nt (56 aa) | 1 + | 73 / 243 |  | - 8.222 |  | 0.788 | - 0.244 | 0.004 | NO |
|  |  | 2 | 132 nt (43 aa) | 1 + | 394 / 525 |  |  |  |  |  |  |  |
