# Supplementary figures and images for: MAGI2 Gene Region and Celiac Disease
Source: Front Nutr. 2019 Dec 19;6:187. doi: 10.3389/fnut.2019.00187 (PMC6930898; doi:10.3389/fnut.2019.00187)

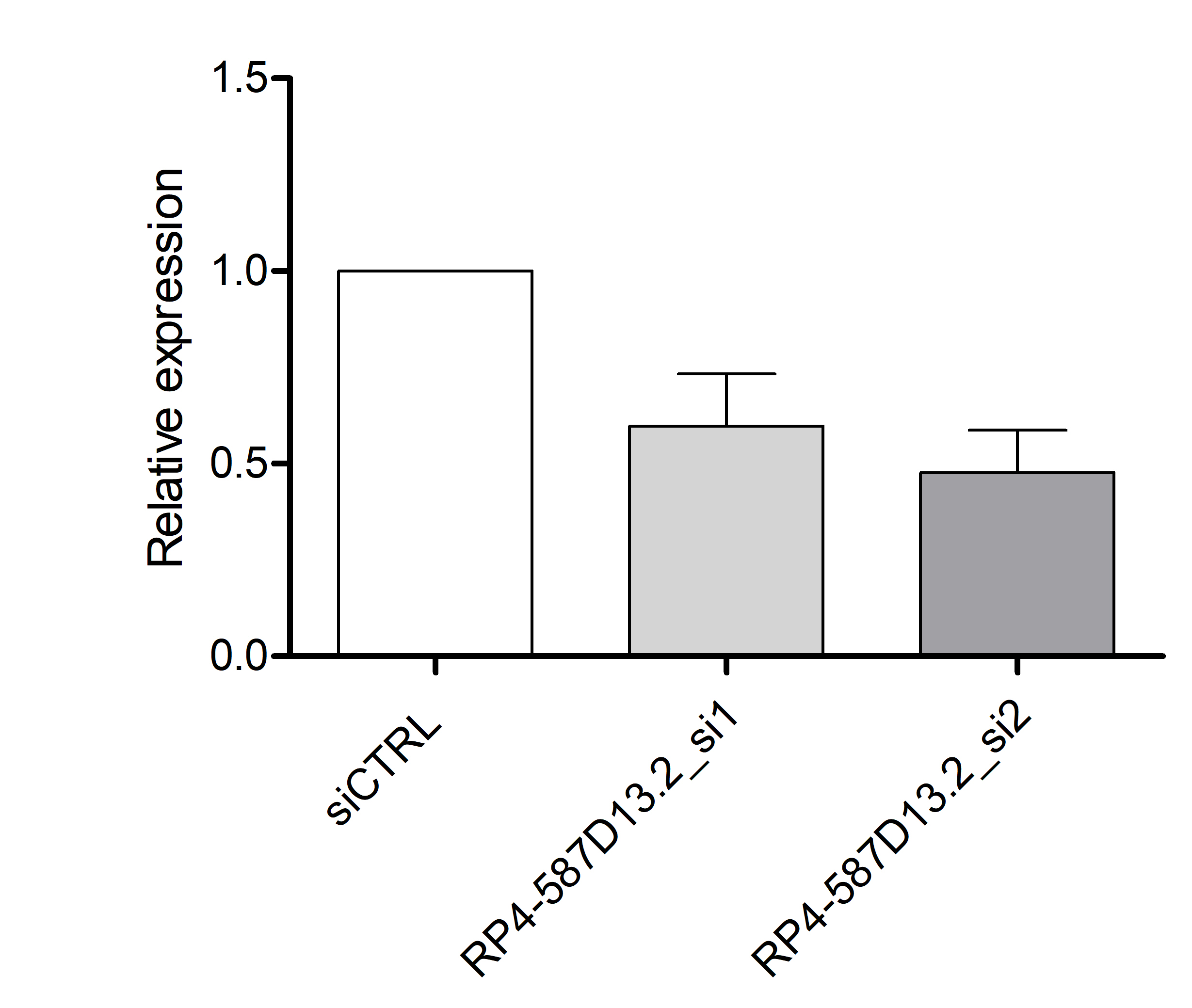

Supplement: Supplementary file 1 [file Data_Sheet_1.ZIP › Supplementary material/Figures/Figure S1 .jpg]

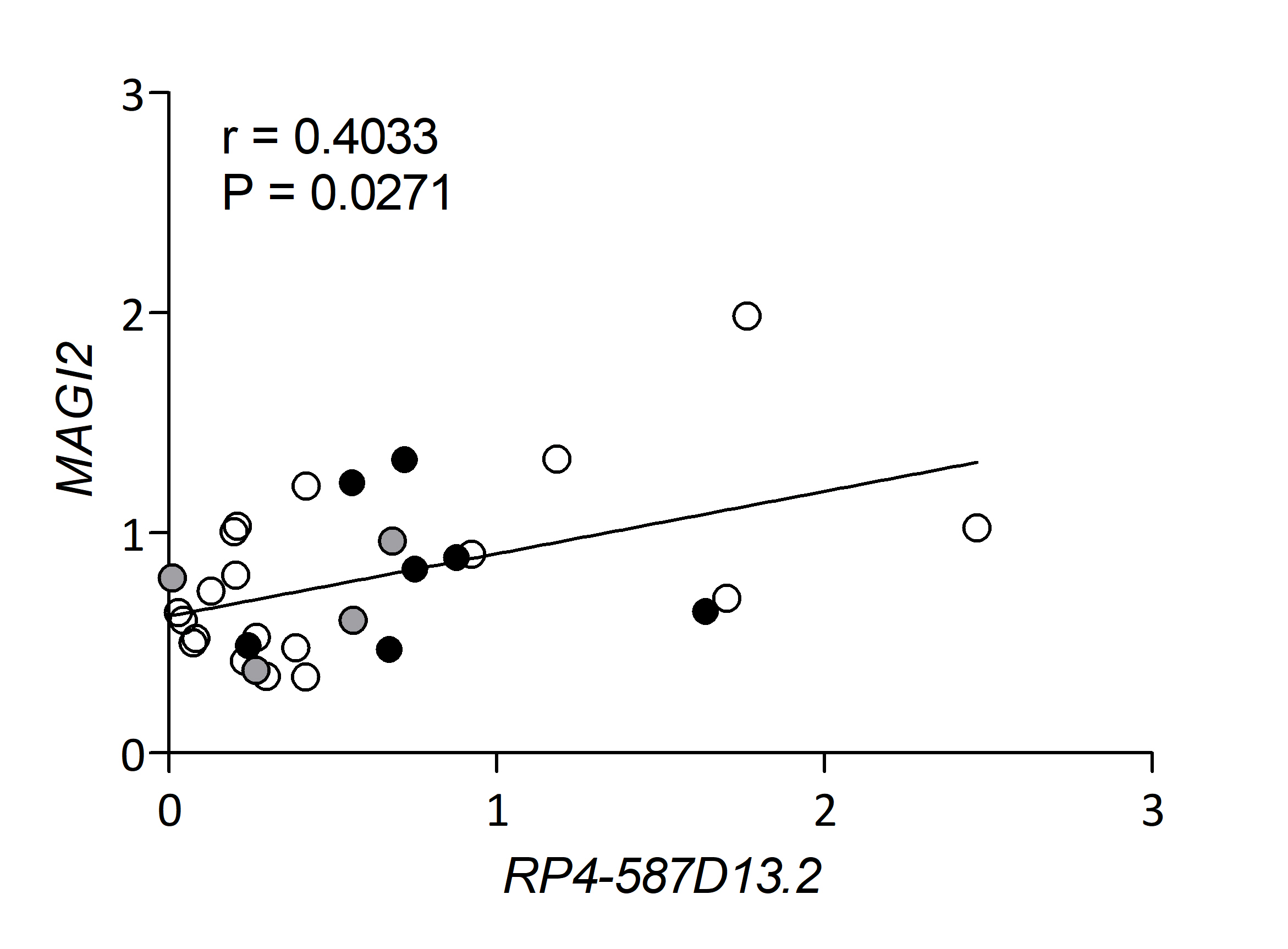

Supplement: Supplementary file 1 [file Data_Sheet_1.ZIP › Supplementary material/Figures/Figure S2.jpg]
